# Influence of Sex on the Recovery Response Following Isometric Exercise Performed at Long Muscle Length

**DOI:** 10.1519/JSC.0000000000005521

**Published:** 2026-05-12

**Authors:** Giuseppe Rosaci, Federico Nigro, Samuele M. Marcora, Sandro Bartolomei

**Affiliations:** Department for Life Quality Studies, University of Bologna, Bologna, Italy

**Keywords:** muscle thickness, muscle echo-intensity, strength, pushing isometric muscle action, muscle fatigue

## Abstract

Rosaci, G, Nigro, F, Marcora, SM, and Bartolomei, S. Influence of sex on the recovery response following isometric exercise performed at long muscle length. *J Strength Cond Res* 40(10): e1039–e1045, 2026—The aim of the study was to compare the recovery responses of the pectoralis major muscle in men and women after isometric exercise. Twelve men (age: 25.3 ± 4.1 years; height: 177.6 ± 7.4 cm; body mass: 81.6 ± 10.1; training experience: 6.7 ± 3.7) and 10 women (age: 24.5 ± 1.4 years; height: 166.4 ± 6.7 cm; body mass: 62.5 ± 7.4; training experience: 5.0 ± 2.7) performed a high-intensity (maximum effort) intermittent isometric exercise at long muscle length (LML) for the pectoralis major muscle. Peak force at LML and short muscle length, average power at bench throw power test, muscle architecture (muscle thickness and corrected echo intensity), and muscle soreness were assessed before, and 15 minutes, 24 and 48 hours postexercise. Rate of perceived exertion was also collected 15 minutes postexercise. Although men were significantly stronger that women, no sex differences were detected in changes of peak force at LML (*p ≥* 0.184) and short muscle length (*p ≥* 0.514), muscle architecture (*p ≥* 0.552), soreness (*p ≥* 0.09), and on rate of perceived exertion (*p* ≥ 0.452). However, a greater drop in average power at the bench throw power test was registered in men compared with women 15 minutes postexercise (*p* < 0.001, *d* = 2.36). Although similar patterns of muscle recovery were observed in men and women, a sex-related difference may exist in the acute effects of isometric exercise on power exertion. Men indeed may be more sensitive to acute fatigue and more prone to power reductions after isometric exercise.

## Introduction

Isometric exercise is considered an effective method to elicit neuromuscular adaptations and changes in muscle architecture in the different populations of practitioners and athletes (37). Parameters such as contraction duration (time under tension), training volume, intensity, and muscle length represent key factors that can influence adaptations promoted by isometric training strategies. In particular, the length of the target muscles during isometric contractions represents a variable that should be considered. Greater effects on muscle hypertrophy (2), and peak force (41) indeed, were reported when isometric contractions were performed at long muscle length (LML) compared with short muscle length (SML).

Muscle length may also affect the recovery process after isometric exercise and, as a consequence, long-term muscle adaptations (19). Greater and more prolonged declines in peak force were reported after isometric exercises performed at LML compared with SML in knee extensors (31) and elbow flexor muscles (3). However, the previous studies were conducted on male individuals only, and sex may influence the magnitude of performance decline and the time course of muscle recovery after isometric exercise (29).

Some authors indeed, registered a lower fatigability in women compared with men, during prolonged-low intensity isometric contractions of the knee-extensor and elbow flexor muscles (6,24,42). On the contrary, studies comparing the recovery responses after traditional resistance exercise protocols in men and women are still inconclusive and contradictory. Lewis et al. were unable to detect any difference up to 48 hours after a high volume resistance exercise session (27), while Judge and Burke reported a faster recovery rate in women compared with men, following a high-intensity bench press protocol (23). Thus, current evidences do not consent to draw conclusions about the differences in the recovery process after resistance exercise between men and women (4) and in particular after high-intensity isometric exercise. To the best of our knowledge indeed, no studies have investigated sex differences in the recovery response after a high-intensity intermittent isometric exercise session focused on upper-body muscles.

Therefore, the primary aim of this study was to examine the changes in strength, average power, muscle architecture, and soreness after an isometric exercise session performed at LML in trained men and women. The authors hypothesized that greater reductions in strength and power, and more pronounced acute changes in muscle architecture may occur in men compared with women.

## Methods

### Experimental Approach to the Problem

**Figure 1.**
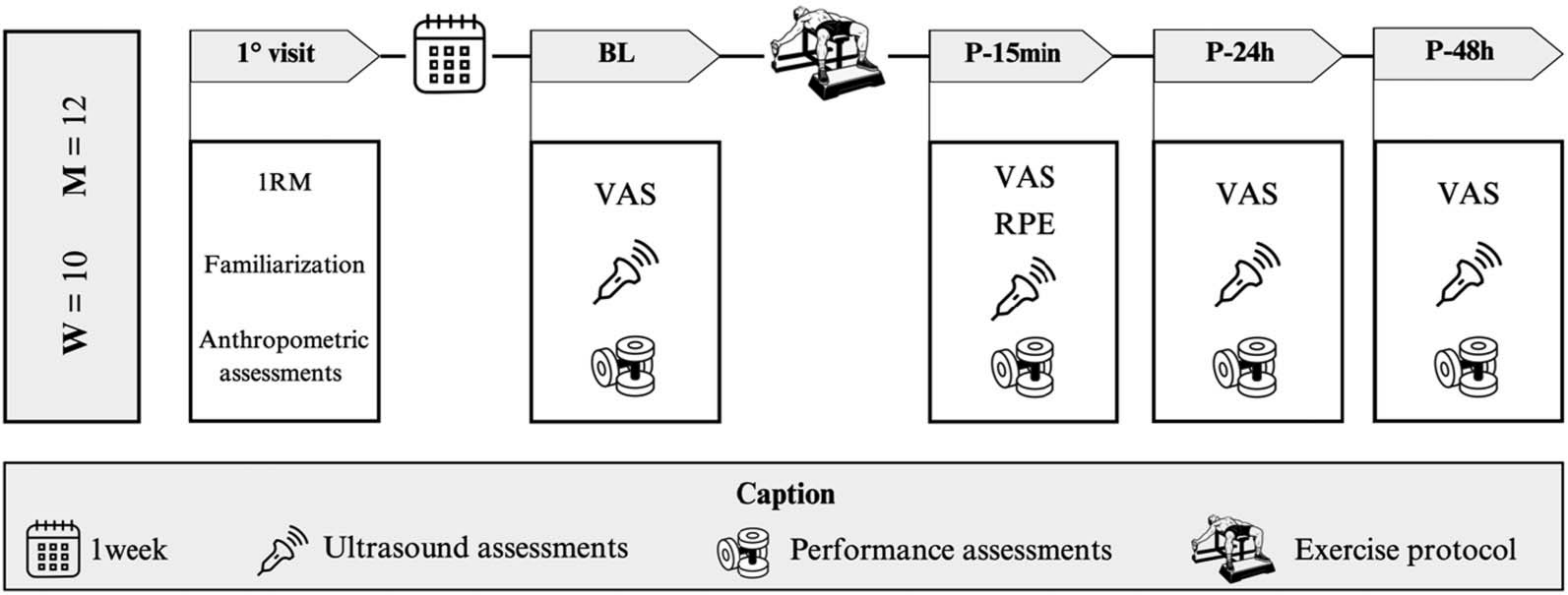
Experimental design of the study. 1-RM = 1 repetition maximum in bench press exercise; VAS = Visual Analog Scale; RPE = rate of perceived exertion; BL = baseline; P-15min = 15 minutes post exercise; P-24h = 24 hours post exercise; P-48h = 48 hours post exercise. M = men; W = women.

A single-intervention repeated-measures design, consisting of 4 experimental visits to the human performance laboratory, was used (Figure 1). The first visit included the assessment of anthropometric measurements (body mass, height, and arm length), the determination of the one-repetition maximum (1-RM) at the bench press exercise, and the familiarization with the isometric exercises and with the bench press throw power test. Each subject returned to the laboratory 1 week after their initial visit to perform the isometric exercise protocol. Subjects were tested for muscle architecture, isometric peak force at LML and SML, average power and muscle soreness before and 15 minutes (P-15min), 24 hours (P-24h), and 48 hours (P-48h) after the intervention. The rate of perceived exertion (RPE) was assessed at P-15min, only.

### Subjects

A priori power analysis was conducted using G*Power 3.1.9.7 to determine the required sample size for detecting a Sex × Time interaction in a repeated-measures design using linear mixed models. Parameters used were: effect size *f* = 0.30, *α* = 0.05, power = 0.80, 2 groups, 4 measurements (Baseline, P-15min, P-24h, and P-48h), correlation among measures = 0.5, nonsphericity correction *ε* = 0.75. The required sample consisted of 18 subjects. Inclusion criteria required subjects to be between the age of 18 and 35 years, with a minimum of 2 years of resistance training experience and without injuries in the 12 months before the starting of the study. Twelve resistance-trained (Tier 2: trained/developmental) men (age: 25.3 ± 4.1 years; height: 177.6 ± 7.4 cm; body mass: 81.6 ± 10.1; training experience: 6.7 ± 3.7; arm length: 65.5 ± 3.4 cm; 1-RM bench press: 108.5 ± 21. kg) and 10 resistance-trained (Tier 2: trained/developmental) women (age: 24.5 ± 1.4 years; height: 166.4 ± 6.7 cm; body mass: 62.5 ± 7.4; training experience: 5.0 ± 2.7; arm length: 59.7 ± 2.3 cm; 1-RM bench press: 47.6 ± 13.1 kg) volunteered to participate in this study (30). The phase of the menstrual cycle in which the female subjects were tested was not considered in this study. Women participating in this investigation were not using any hormonal contraceptives. All the subjects signed an informed consent, and the study was approved by the local Bioethic Committee (n. 0070230). Subjects were asked to abstain from physical activity for the 48 hours before the beginning of the experiment. No alcohol, caffeine, or supplements (i.e., creatine) were allowed during the entire intervention.

### Strength and Power Assessments

**Figure 2.**
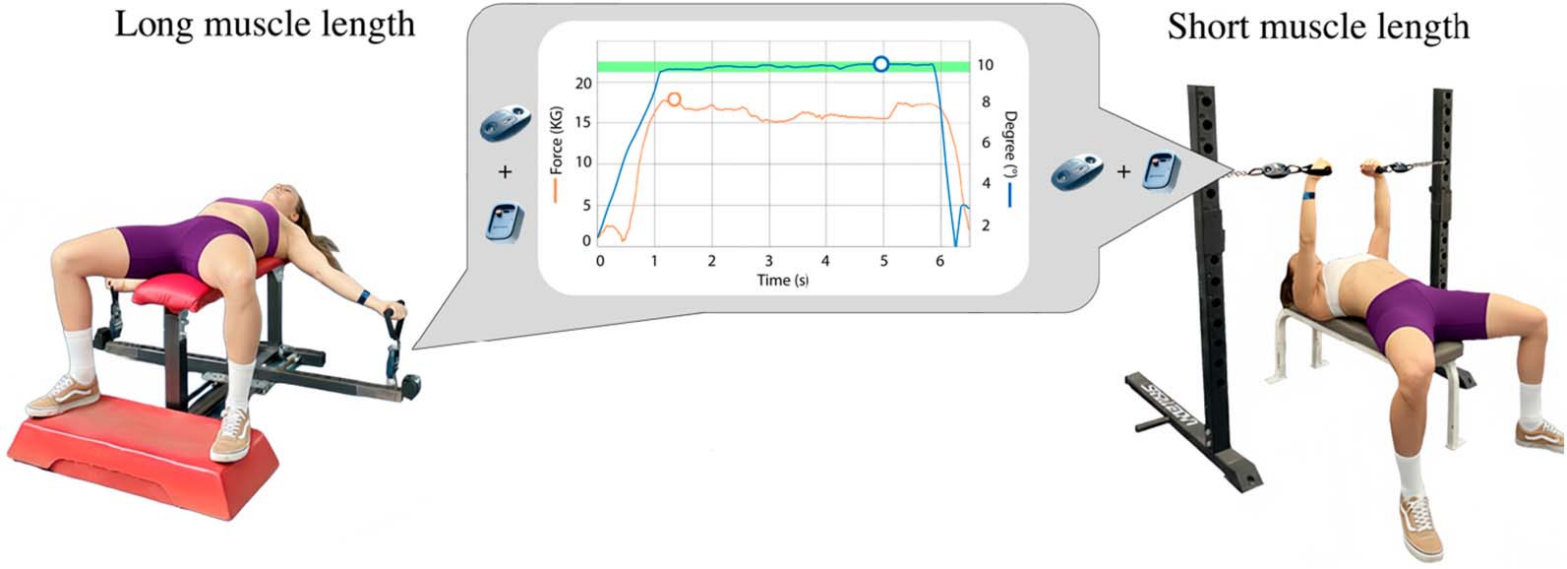
Experimental setup (body position, synchronized dynamometer and goniometer signals) for testing and exercise at long muscle length and for testing only at short muscle length.

A standardized warm-up, previously described by Bartolomei et al. (10), was performed before each testing session. In the first visit, the 1-RM bench press was assessed as described by Hoffman (21). Trials not meeting the range of motion criteria for each exercise or where technique was not appropriate were discarded. A bench throw power test (BTP) was performed to assess the average power at each time point using a load equal to the 30% of the previously tested 1-RM. During the BTP test, subjects were instructed to push as explosively as possible until full arm extension and to project the bar as high as possible. No stretch-shortening cycle action was involved, and the barbell was pushed upward directly from a static position (barbell in contact with the chest). A flat scapular position was required to optimize power output. (8). Two spotters were placed at each side of the Smith machine to decelerate the bar during the descending phase. Two sets of 2 repetitions were required with a recovery time of 20 seconds between each repetition and a 3-minute recovery time was used between each set (12). A linear encoder (Tendo Unit model V104; Tendo Sports Machines, Trencin, Slovak Republic) was used to measure the average power during all BTP repetitions. The intraclass correlation coefficient (ICC) for the BTP was 0.97 (SEM: 13.1 W). At each time points (BL, P-15min, P-24h, and P-48h), subjects completed a maximum isometric force test for the pectoralis major muscle performed at LML and SML, in a randomized order. For the LML assessments, a custom-made apparatus consisting of a horizontal bench provided by 2 extendable arms positioned at the floor level was used. A wireless dynamometer (Kinvent Physio, Montpellier, France, sampling frequency of 1,000 Hz) was attached to the end of each extendable arm to measure peak force. The LML isometric test was performed with the body in supine position and the shoulder abducted at −45° (corresponding to the end of the eccentric phase of the chest fly exercise). The isometric test at SML was performed on a power rack, with the subject in supine position and the arms perpendicular to the ground. During all the isometric assessments and the subsequent exercise protocols, the elbow was maintained at 10° of flexion and monitored using a digital goniometer (K-Move, Kinvent Physio, Montpellier, France) (Figure 2). Subjects were asked to pull as hard and as fast as possible (20) for 6 seconds and the isometric peak force was registered. The same qualified investigators supervised the testing sessions and the exercise protocol, and the subjects were verbally encouraged. The ICCs were 0.90 (SEM: 14.20 N) and 0.94 (SEM: 11.0N) for LML peak force and SML peak force, respectively.

### Exercise Protocols

The isometric exercise protocol consisted of 6 sets of 5 pushing isometric muscle actions lasting for 5 seconds, with 5 seconds of rest between the contractions and 75 seconds of rest between the sets, performed at LML. LML exercise protocol reproduced the setup adopted for the LML isometric peak force assessment. Similarly, isometric peak forces and angles for each isometric contractions of the exercise protocol were measured using the aforementioned wireless dynamometer and digital goniometer. Subjects received continuous visual and verbal feedback during the exercise protocol. Percentage decrease in peak force between the first and the last repetition, and the percentage decrease in the average peak force of the first and last sets of the protocol were calculated as fatigue indexes. In addition, fatigue dissipation (percentage change in force between the last repetition of the preceding set and the first repetition of the subsequent set) was calculated.

### Ultrasound Measurements

Before the warm-up, subjects were asked to lie down in a supine position on a physiotherapy bed relaxing their arm and chest muscles for at least 15 minutes before ultrasound measurements. Skeletal muscle ultrasound images were collected from the subject's right side using a 12-MHz linear probe (Echo Wave 2, Telemed Ultrasound Medical System, Milan, Italy) coated with water-soluble transmission gel at BL, P-15min, P-24h, and P-48h. The probe was positioned on the skin surface without depressing the dermal layer, and the view mode (gain = 125 dB; image depth = 5 cm) was used to obtain images of the pectoralis major muscle. Three consecutive images were acquired and analyzed by the same technician. The pectoralis muscle thickness (MT) was measured at the site between the third and fourth costa below the center of the clavicle (1). For each image, MT was measured with a single perpendicular line from the superficial aponeurosis to the deep aponeurosis (34), and subcutaneous fat thickness was quantified as the distance between the skin–muscle interface and the superior border of the muscle's aponeurosis (39). The average of 3 MT measurements was used for statistical analysis. The ICC was 0.95 (*SEM* = 1.05 mm). The images were imported into a specific software (ImageJ, version 1.53k, National Institutes of Health, USA) in .TIF format. Pectoralis muscle echo-intensity (EI) was calculated using the polygon selection tool (26) in the largest possible region of interest across all ultrasound images. In addition, the raw EI was corrected for subcutaneous fat using the following equation (43):

Corrected EI (cEI)=Raw EI+(Subcutaneous fat×40.5278).

### Muscle Soreness and Perceived Effort

Fifteen minutes after completing the exercise protocol, subjects were asked to answer to the following question: “How was your workout?” using a rating of perceived exertion (RPE) scale, where 0 = very easy, 2 = easy, 3 = moderate, 4 = fairly hard, 5–6 = hard, 7–9 = very hard, and 10 = maximum (18). In addition, muscle soreness of the pectoralis major muscle was assessed using a 100-mm visual analog scale, where 0 indicated no pain and 100 indicated the worst pain imaginable (16). Soreness intensity was measured at BL and repeated at each time point following the exercise protocol.

### Statistical Analyses

Descriptive statistics were reported as means and standard deviations (*SD*). Independent sample *t*-tests were conducted to assess sex-based differences in individual variables at BL (peak forces, average power at BTP, MT, and cEI), exercise-related outcomes (percentage changes in peak force), and the RPE registered following the exercise session. In addition, linear mixed models were adopted to assess recovery over time between men and women, and a different model was used for each variable (dependent). The models were specifically designed to test the Sex × Time interaction as the primary outcome, where “Sex,” “Time,” and “BL values” were used as fixed factors while “subjects” was set as a random effect. This approach was adopted to consider the differences between the subjects at baseline. In addition, significant main effects were examined using simple main effect analyses and pairwise comparisons were performed using with Tukey's post hoc test. ANOVA *F*-statistics were converted to partial eta squared (*η*_*p*_^2^) along with their 95% confidence intervals (95% CI). The effect sizes were interpreted as: ≤0.01 (trivial), >0.01–0.06 (small), >0.06–0.14 (moderate), and >0.14 (large) (Cohen, 1988). Similarly, t-statistics from LMMs were converted into Cohen's *d* effect sizes with associated 95% CIs, and interpreted as: ≤0.2 (trivial), >0.2–0.6 (small), >0.6–1.2 (moderate), >1.2–2.0 (large), >2.0–4.0 (very large), and ≥4.0 (extremely large). Statistical significance was set at *p* ≤ 0.05. All the analyses were performed using R software (R Foundation for Statistical Computing, Vienna, Austria).

## Results

All the data relative to the exercise protocol, performance assessments, muscle architecture and muscle soreness are summarized in Tables 1–3.

**Table 1:** Data relative to the isometric exercise protocol at long muscle length in men and women.

Parameters	Intervals	Men	Women
Peak force (N)	1st repetition	166.7 ± 42.1	93.9 ± 19.4
	30th repetition	117.8 ± 26.8	67.0 ± 14.8
	1st set	150.5 ± 39.8	84.9 ± 14.1
	6th set	129.5 ± 27.9	69.1 ± 14.1
Fatigue indices (%)	1st to 30th repetition	−27.9 ± 15.7	−26.8 ± 18.7
	1st to 6th set	−12.4 ± 14.4	−18.5 ± 10.6
Recovery between sets (%)	1st to 2nd set	8.4 ± 15.4	6.6 ± 17.4
	2nd to 3rd set	17.5 ± 16.5	8.1 ± 14.9
	3rd to 4th set	10.1 ± 18.6	10.7 ± 22.0
	4th to 5th set	27.6 ± 15.8	8.0 ± 13.7
	5th to 6th set	28.2 ± 16.1	22.1 ± 9.0

Indicates a significant difference between sexes.

**Table 2:** Data relative to performance assessments, muscle architecture, muscle soreness, and RPE registered at the different timepoints following exercise protocol in men (M) and women (W).^*^

	Assessments	Sex	BL	P-15min	P-24h	P-48h
Performance	Peak force at LML (N)	M	194.4 ± 49.2	171.9 ± 32.3†	184.2 ± 34.9	190.2 ± 39.7
		W	91.1 ± 18.9	85.1 ± 17.2†	87.4 ± 21.4	86.4 ± 20.9
	Peak force at SML (N)	M	207.4 ± 44.2	196.2 ± 33.1†	201.7 ± 38.4	213.1 ± 43.3
		W	99.7 ± 19.1	90.8 ± 19.7†	96.3 ± 20.6	97.9 ± 17.6
	Average power at BTP (W)	M	449.1 ± 85.9	425.4 ± 82.4†,‡	445.5 ± 81.0	449.2 ± 78.6
		W	198.5 ± 26.3	188.7 ± 28.6†	197.8 ± 26.6	194.0 ± 28.4
Ultrasound	MT (mm)	M	2.11 ± 0.46	2.23 ± 0.38†	2.15 ± 0.44†	2.08 ± 0.41
		W	0.93 ± 0.13	1.06 ± 0.16†	1.03 ± 0.18†	0.93 ± 0.12
	cEI (au)	M	66.9 ± 11.10	69.98 ± 9.02	67.13 ± 12.99	65.66 ± 10.2
		W	90.3 ± 22.48	92.62 ± 19.21	89.64 ± 19.05	84.52 ± 18.30
Soreness and perception	VAS (mm)	M	0.0 ± 0.0	42.29 ± 23.7	18.42 ± 14.4	6.58 ± 10.1
		W	0.0 ± 0.0	44.6 ± 27.53	38.20 ± 22.70	28.40 ± 24.99
	RPE	M	—	7.21 ± 1.81	—	—
		W	—	7.10 ± 0.77	—	—

* LML = long muscle length; SML = short muscle length; BTP = bench throw power test; MT = pectoralis major muscle thickness; cEI = corrected echo-intensity for pectoralis major muscle; VAS = Visual Analogue Scale. RPE = rate of perceived exertion; BL = baseline; P-15min = 15 min post exercise; P-24h = 24 h post exercise; P-48h = 48 h post exercise.

†Indicates a significant difference from baseline (main effect of time with group combined).

‡ Indicates a significant sex × time interaction.

**Table 3:** Percentage changes relative to performance assessments and muscle architecture registered at the different timepoints following exercise protocol in men (M) and women (W).*†

	Assessments	Sex	BL	P-15min	P-24h	P-48h
Strength and power	Peak force at LML (%)	M	0 ± 0	−9.8 ± 11.3	−3.5 ± 10.3	0.6 ± 20.6
		W	0 ± 0	−6.2 ± 8.6	−4.2 ± 13.0	−4.5 ± 10.6
	Peak force at SML (%)	M	0 ± 0	−4.2 ± 10.7	−1.8 ± 10.4	3.9 ± 14.2
		W	0 ± 0	−9.2 ± 7.6	−3.3 ± 11.5	−0.4 ± 14.4
	Average power at BTP (%)	M	0 ± 0	−5.2 ± 5.1	−0.5 ± 4.1	0.4 ± 5.2
		W	0 ± 0	−5.2 ± 3.5	−0.3 ± 3.0	−2.3 ± 3.4
Muscle architecture	MT (%)	M	0 ± 0	−7.1 ± 8.3	−2.5 ± 4.2	−1.0 ± 4.0
		W	0 ± 0	−13.9 ± 10.6	−9.6 ± 8.1	−0.1 ± 5.4
	cEI (%)	M	0 ± 0	−5.8 ± 8.2	0.5 ± 6.6	−1.2 ± 4.8
		W	0 ± 0	−4.1 ± 11.8	0.6 ± 12.8	−4.2 ± 16.2

LML = long muscle length; SML = short muscle length; BTP = bench throw power test; MT = pectoralis major muscle thickness; cEI = corrected echo-intensity for pectoralis major muscle; BL = baseline; P-15min = 15 min post exercise; P-24h = 24 h post exercise; P-48h = 48 h post exercise.

* Indicates a significant difference from baseline (main effect of time with group combined).

† Indicates a significant sex × time interaction.

### Exercise Protocol

**Figure 3.**
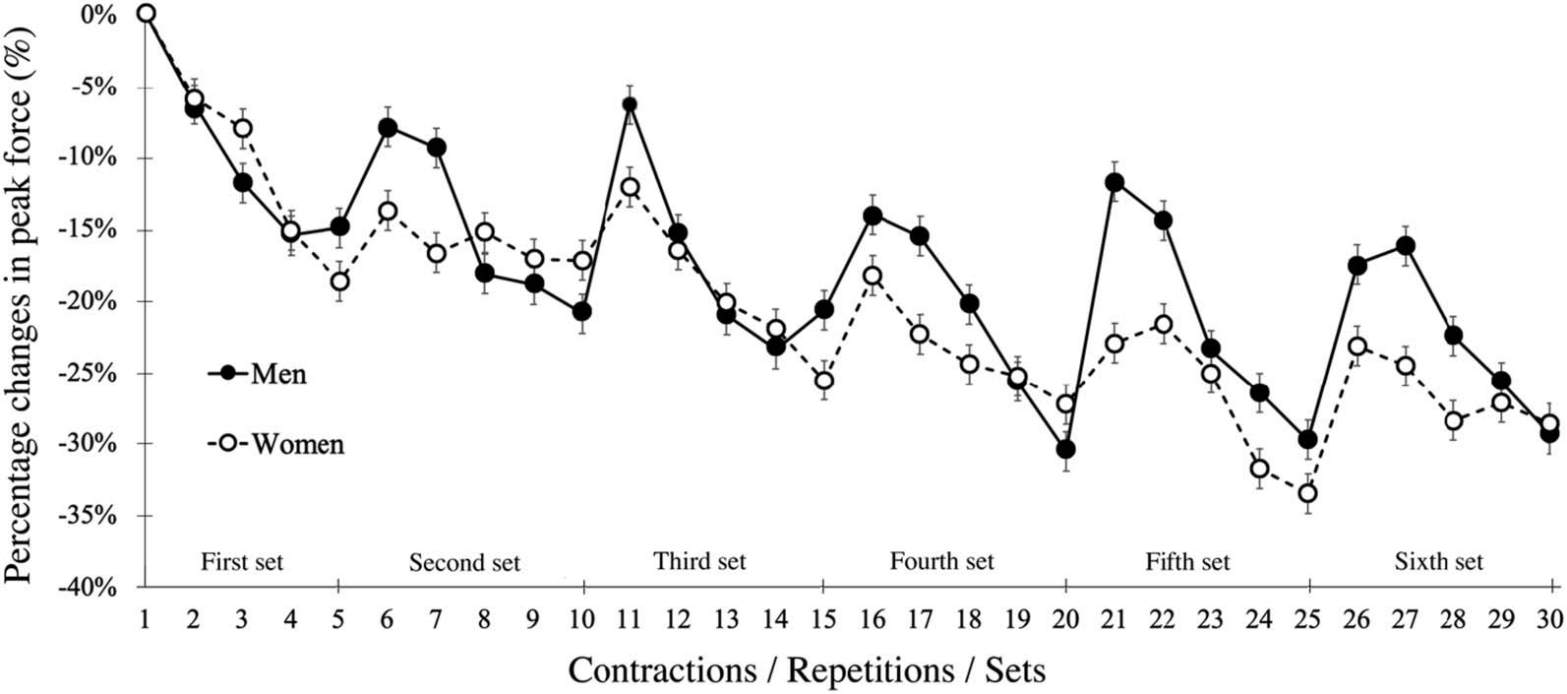
Percentage changes in peak force during the isometric exercise protocol in men and women across all contractions/repetitions.

Men produced higher peak force at LML (192.21 ± 93.46 N vs. 92.67 ± 18.59 N; *p* < 0.001; *d* = 3.052), peak force at SML (206.43 ± 40.05 N vs. 100.01 ± 19.42 N; *p* < 0.001; *d* = 3.280), and average power at BTP (448.83 ± 82.69 W vs. 202.85 ± 28.72 W; *p* < 0.001; *d* = 3.82) compared with women. In addition, higher MT (2.11 ± 0.45 cm vs. 0.94 ± 0.12 cm; *p* < 0.001; *d*: 0.94) and a lower cEI (66.57 ± 9.72 au vs. 90.66 ± 17.10 au; *p* < 0.001; *d*: 0.58) were detected in men compared with women at BL. No sex differences were found in between-contractions fatigue index (*p* = 0.86; *d* = −0.07), between-sets fatigue index (*p* = 0.27; *d* = 0.49) (Figure 3), or intersets fatigue dissipation (*p* > 0.19; *d* > −0.02).

### Strength and Power Assessments

**Figure 4.**
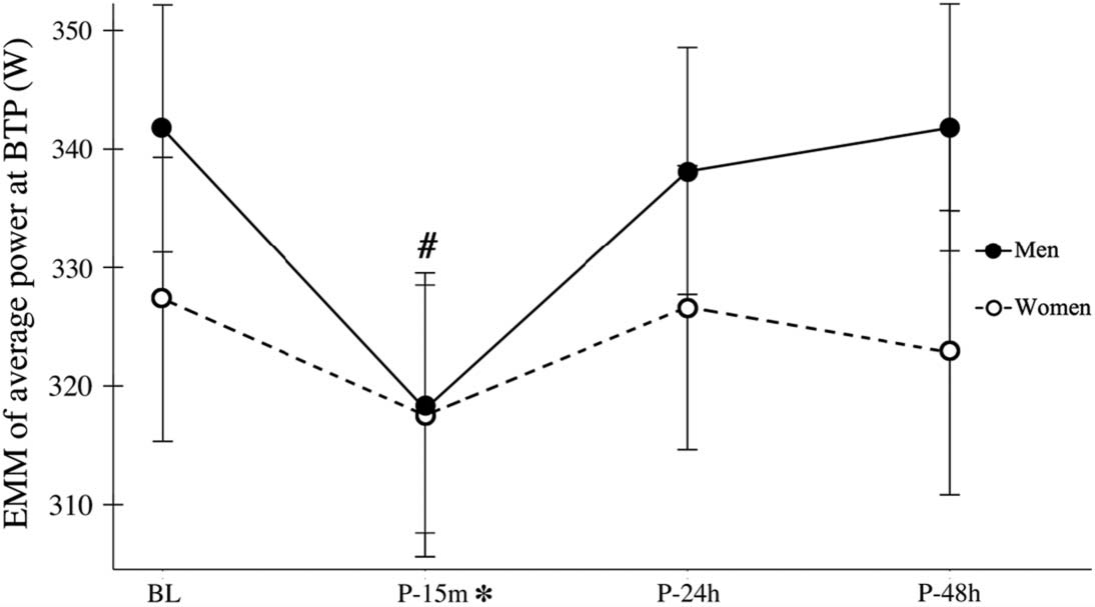
Changes in average power at bench throw power test (BTP) performance at the different timepoints following the exercise protocol in men and women. EMM = estimated marginal means; BL = baseline; P-15min = 15 minutes post exercise; P-24h = 24 hours post exercise; P-48h = 48 hours post exercise. “*” indicates a significant difference from baseline (with group combined). “#” indicates a significant sex × time interaction.

Significant main effects of time were detected for peak force at LML (*p* = 0.009; *F* = 4.224, *η*_*p*_^2^ = 0.18; 95% CI, 0.03–1.00), peak force at SML (*p* = 0.009; *F* = 4.144, *η*_*p*_^2^ = 0.17; 95% CI, 0.03–1.00), and average power at BTP following the exercise protocol (*p* < 0.001; *F* = 12.918; *η*_*p*_^2^ = 0.39; 95% CI, 0.22–1.00). Differences from baseline were significant at P-15min for peak force at LML (*p* = 0.006; *t* = 3.449, *d* = 1.04; 95% CI, 0.44–1.65), peak force at SML (*p* = 0.041; *t* = 2.715, *d* = 0.82; 95% CI, 0.22–1.43), and average power at BTP (*p* < 0.001; *t* = 5.516, *d* = 1.669; 95% CI, 1.06–2.28). No sex × time interactions were found for peak force at LML (*p* = 0.184; *F* = 1.664; *η*_*p*_^2^ = 0.08; 95% CI, 0.00–1.00) and peak force at SML (*p* = 0.514; *F* = 0.773; *η*_*p*_^2^ = 0.04; 95% CI, 0.00–1.00). Conversely, a sex × time interaction was found for average power at BTP (*p* = 0.025; *F* = 3.343, *η*_*p*_^2^ = 0.14; 95% CI, 0.01–1.00). Post hoc comparisons revealed a significant decrease in average power in BTP at P-15min in the men group (−5.2%; *p* < 0.001; *t* = 5.785; *d* = 2.36; 95% CI, 1.55–3.18), only (Figure 4).

### Ultrasound Measurements

A main effect of time was detected for MT (*p* < 0.001; *F* = 17.579, *η*_*p*_^2^ = 0.47; 95% CI, 0.30–1.00) with differences from BL at P-15m (*p* < 0.001; *t*: −5.669, *d* = −1.72; 95% CI, –2.32 to −1.11]) and P-24h (*p* = 0.0170; *t*: −3.059, *d* = −0.93; 95% CI, –1.53 to −0.32]). In addition, a main effect of time was also detected for cEI (*p* = 0.017; *F* = 3.664, *η*_*p*_^2^ = 0.15; 95% CI, 0.02–1.00), but no differences from BL were detected at any timepoints. Conversely, no sex × time interactions in MT (*p* = 0.671; *F* = 0.518, *η*_*p*_^2^ = 0.03; 95% CI, 0.00–1.00) and cEI (*p* = 0.599; *F* = 0.627, *η*_*p*_^2^ = 0.03; 95% CI, 0.00–1.00) were found.

### Muscle Soreness and RPE

A significant main effect of time was detected (*p* < 0.001; *F* = 22.643, *η*_*p*_^2^ = 0.53; 95% CI, 0.38–1.00), with a significant difference from BL at P-15m (*p* < 0.001; *t* = −7.992, *d* = −2.42; 95% CI, –3.03 to −1.81), P-24h (*p* < 0.001; *t* = −5.208, *d* = −1.58; 95% CI, –2.18 to −0.97), and P-48h (*p* = 0.011; *t* = −3.298; *d* = −0.97; 95% CI, –1.58 to −0.37). Finally, no significant differences between the sexes were identified in the RPE at P-15m (*p* = 0.863; *F* = 0.031, *η*_*p*_^2^ = 0.002). No significant sex × time interactions were detected for visual analog scale following the exercise protocol (*p* = 0.097; *F* = 2.204, *η*_*p*_^2^ = 0.010; 95% CI, 0.00–1.00W).

## Discussion

This study investigated sex-related differences in the recovery responses after a high-intensity intermittent isometric exercise performed at LML. Despite the results of this study confirmed previously reported baseline sex differences in maximal force and muscle architecture, our study hypothesis was only partially supported.

As previously reported by many studies (9,11), women typically exhibit significantly lower levels of maximal force, muscle size, and muscle quality, influenced in part by sex hormones, intramuscular fat, and individual fat distribution (17,33). Although these differences exist, peak force, muscle architecture, and muscle soreness followed similar recovery time courses in both sexes. However, the wide confidence intervals (0.00–1.00) may indicate a considerable statistical uncertainty. Therefore, no definitive conclusions regarding sex differences in the recovery time course of these parameters can be drawn, and these findings should be considered as preliminary.

A greater reduction in the upper-body power was detected in men compared with women at P-15min only. This is partially consistent with Amdi et al. (5) who reported a greater drop in barbell peak velocity in men compared with women, 5-minute, 24-hours, and 48-hours after a back squat protocol performed at 80% of 1-RM. Not consistently, Margoni et al. (28) found no sex-related differences in recovery up to 72 hours after a power, strength, or an high-volume resistance training session. It is important to note, however, that Margoni et al. (28) monitored the neuromuscular recovery using maximal isometric tests only and did not include any assessment of power. Dynamic tests are generally considered more sensitive to fatigue and to exercise-induced muscle damage than isometric assessments (15). In previous studies, the reduction of upper-body average power following high-volume resistance exercise protocols has been associated to increased muscle thickness (13,14). Our study indicated that isometric exercise may affect muscle thickness up to 24-h after the workout (*p* = 0.017; *t*: −3.06, *d* = −0.93; 95% CI, –1.53 to −0.32]) in both groups. Thus, the greater decrease in average power registered in men 15-minute after exercise may be attributed to a greater supraspinal fatigue detected in men compared with woman after isometric exercise (40). In addition, differences between the sexes in the stiffness of the muscle-tendon unit may have influenced the drop in average power. Men indeed are generally characterized by higher percentages of type II fibers (35) and by a higher tendon stiffness compared with women (36). Conversely, the higher tendon compliance generally observed in women (25) may have attenuated the mechanical stress transmitted to the muscle fascicles and to the sarcomeres during the isometric exercise.

Similar decreases in fatigue indices were observed in both sexes. This is consistent with previous investigations, that reported similar declines in force in men and women during a sustained 60-second maximal isometric contraction (40). Furthermore, these finding are supported by the absence of differences in RPE between the 2 groups. The lack of differences between the sexes in the fatigue indices may be related to a similar vascular compression and reduction in blood flow during muscle contraction, especially when the isometric contractions are performed with maximum effort (7,22).

A possible limitation of this study may be represented by the absence of evaluation of the menstrual cycle phase in the women's group. Although many research reported nonsignificant variations in strength and power performances in the different phases of the menstrual cycle (38), muscle adaptations and recovery may be influenced by this parameter (32). In addition, although the study was powered to detect a medium-to-large effect (*f* = 0.30), it may not have been sufficiently sensitive to identify smaller, more subtle interaction effects. In conclusion, men and women seem to exhibit similar time-course of isometric strength, muscle architecture and muscle soreness following a high-intensity isometric exercise protocol performed at LML. Only power at BPT 15min after the exercise protocol was lower in men, suggesting that sex differences in fatigue may be task-specific and present only very acutely.

**Practical Applications**

The current outcomes may have some interesting implications on the practice of resistance training in men and women. Although similar patterns of muscle recovery were observed in men and women, a sex-related difference may exist in the acute effects of isometric exercise on power exertion. Because the effectiveness of power training is enhanced by a high neuromuscular activation and impaired by fatigue, complex isometric protocols may not be recommended before ballistic or plyometric exercises, in particular in male athletes. As recovery generally seemed to be completed within 24 hours post-PIMA exercise, coaches may take this into account when planning subsequent training sessions.
